# Phase I, two-way, crossover study to demonstrate bioequivalence and to compare safety and tolerability of single-dose XM17 vs Gonal-f^®^ in healthy women after follicle-stimulating hormone downregulation

**DOI:** 10.1186/s12958-015-0124-y

**Published:** 2015-12-01

**Authors:** Andreas Lammerich, Arnd Mueller, Peter Bias

**Affiliations:** Merckle GmbH, Member of the Teva Group, Graf-Arco-Str. 3, 89079 Ulm, Germany

**Keywords:** Recombinant human follicle-stimulating hormone, Infertility, Pharmacokinetics

## Abstract

**Background:**

XM17 is a recombinant human follicle-stimulating hormone (rhFSH) intended mainly for use in controlled ovarian hyperstimulation and the treatment of anovulation. The purpose of the current study was to establish bioequivalence, safety and tolerability of single 300-IU subcutaneous (sc) doses of XM17 to that of the reference follitropin alfa (Gonal-f^®^) in healthy young women.

**Methods:**

This open-label, Phase I, single-dose, single-center, two-way crossover study was conducted from February to May 2009. Thirty-six women aged 18–39 years were included, with a study duration of ~27 days per participant. After endogenous FSH downregulation with goserelin (3.6 mg) on study Day 0, XM17 and Gonal-f^®^ were administered on Days 11 and 19 in random sequence. Frequent serum samples were drawn for standard pharmacokinetics until 168 h postdosing. Laboratory values, adverse events (AEs) and local tolerability were assessed throughout the study period. Primary endpoints included C_max_ and AUC_0-t_. Secondary endpoints included additional pharmacokinetic (PK) parameters, safety and tolerability.

**Results:**

Ratios of XM17 to Gonal-f^®^ for C_max_ and AUC_0-t_ equaled 1.017 (90 % confidence interval [CI]: 0.958, 1.080) and 1.028 (90 % CI: 0.931, 1.134), respectively, with the CIs contained within the predefined interval (0.8, 1.25). Ratios for AUC_0-168h_, AUC_0-∞_ and t_1/2_ were also ~1, and no difference in t_max_ was detected. Both XM17 and Gonal-f^®^ were well tolerated, with no detectable anti-FSH antibodies, serious AEs or AEs leading to discontinuation or dose reduction.

**Conclusions:**

PK bioequivalence of single 300-IU sc doses of XM17 to the reference product Gonal-f^®^ was statistically demonstrated. XM17 was well tolerated both systemically and locally.

**Trial registration:**

ClinicalTrials.gov: NCT02592031; date of registration: 28 October, 2015.

## Background

Therapeutic proteins manufactured by recombinant DNA methods are commonly used in assisted reproductive technology procedures [1–3]. Recombinant human follicle-stimulating hormone (rhFSH; follitropin) is one such protein used to treat infertility [1]. Follitropin alfa (eg, Gonal-f^®^, Merck Serono, Feltham, UK; Bemfola^®^, Finox Biotech AG, Balzers, Liechtenstein) and follitropin beta (eg, Puregon^®^, Organon, Oss, The Netherlands; Follistim^®^, Merck Sharp & Dohme B.V., Whitehouse Station, NJ, USA) are commercially available. Both types are manufactured in Chinese hamster ovary (CHO) cells into which the human gene for FSH has been inserted.

XM17 (Ovaleap^®^; follitropin alfa, Teva Pharmaceuticals Europe B.V., Utrecht, The Netherlands) is a recombinant follitropin alfa manufactured in CHO cells and intended mainly for use in clinical practice as a daily self-administered injection across multiple days for controlled ovarian hyperstimulation and the treatment of anovulation, as well as in severe FSH or luteinizing hormone deficiency and spermatogenesis stimulation. XM17 has been approved by the European Medicines Agency (EMA) as a biosimilar medicinal product to the biological reference medicine follitropin alfa (Gonal-f^®^). The structure, conformation, impurity profile and potency of XM17 are similar to those of Gonal-f^®^ and the amino acid sequence is identical [4].

The term “biosimilar” has been used to describe “…a copy version of an already authorized biological medicinal product with demonstrated similarity in physicochemical characteristics, efficacy and safety, based on a comprehensive comparability exercise” [5]. Manufacturing of biosimilars is complex and must adhere to strict standards [6, 7]. Their development follows a regulatory pathway involving a comprehensive battery of testing to demonstrate comparability of analytical, bioanalytical, pharmacokinetic (PK), pharmacodynamic (PD), efficacy and safety profiles to a reference standard that is an approved product.

In a previous Phase 1 study, single ascending doses of XM17 up to 300 IU have demonstrated safety, tolerability and dose-proportional PK in healthy young women (*n* = 40) whose endogenous FSH was downregulated with goserelin, a gonadotropin-releasing hormone (GnRH) agonist [8]. This Phase I study was conducted to assess the bioequivalence at a PK level of XM17 to that of the reference follitropin alfa (Gonal-f^®^) in healthy young women.

## Methods

### Ethics, consent and study conduct

This was a Phase I, open-label, randomized, single-dose, two-way crossover study conducted at a single center from February to May 2009 (ClinicalTrials.gov: NCT02592031;+EudraCT Number: 2008-005756-24). The study was approved by the Ethics Committee of the General Medical Council Baden-Württemberg, Germany and conducted according to the ethical principles set forth in the Declaration of Helsinki [9] and applicable Good Clinical Practice Guidelines [10]. Patients provided signed informed consent prior to participation**.**

### Study objectives and parameters measured

The primary objectives were to assess the PK (ie, bioequivalence) and safety in terms of both systemic and local tolerability of single 300-IU sc doses of XM17 (Ovaleap^®^; follitropin alfa, Teva Pharmaceuticals Europe B.V., Utrecht, The Netherlands) and Gonal-f^®^. The primary PK parameters measured for XM17 and Gonal-f^®^ included area under the serum concentration vs time curve (AUC) from time 0 to the last concentration observed above predose levels (AUC_0-t_) and maximum predose-corrected serum concentration (C_max_). Secondary PK parameters included AUC from time 0 to 168 h (AUC_0-168h_), AUC extrapolated to infinity (AUC_0-∞_), apparent terminal rate constant (λ_Z_), apparent volume of distribution during the terminal phase (V_z_/F where F = fraction absorbed), time at which C_max_ occurred (t_max_), apparent terminal elimination half-life (t_1/2_), and apparent clearance (CL/F).

Safety in terms of systemic and local tolerability (ie, injection-site reactions) was monitored in all participants who received at least one dose of either study drug. Treatment-emergent adverse events (TEAEs) and adverse reactions, changes in laboratory parameters (hematology, clinical chemistry, coagulation parameters, urinalysis), changes in vital signs and electrocardiogram (ECG), presence of anti-FSH antibodies and changes in participants’ physical or gynecological examination were collected.

### Participant eligibility

Nonpregnant, non–breast-feeding women aged 18–39 years of any race, with a body mass index (BMI) between 18 and 29 kg/m^2^ and weighing ≥50 kg were included. Participants could be either non- or moderate smokers (<10 cigarettes/day). All must have used oral contraceptives (OCs) for ≥3 months for contraception, had a normal uterus and two functioning ovaries, and agreed to use double-barrier contraceptive methods during the study and resume OCs after end of study. Participants must have been healthy by medical history, physical examination, ECG, blood pressure (BP), pulse rate, blood laboratory profile, including coagulation factors and urinalysis. Participants must have tested negative for HIV and hepatitis C virus, and were excluded if they had a history of or current polycystic ovary syndrome, class III or IV endometriosis, Papanicolaou (PAP) smear ≥3 within 3 years of screening, submucosal myoma uteri, endocrine abnormalities with treatment within 6 months of screening, drug or alcohol abuse, high caffeine consumption (>5 coffee equivalents/day), or treatment with an investigational drug or a drug with known potential organ toxicity within 60 or 90 days, respectively.

### Study assessments and design

The study consisted of a screening visit within 21 days before the start of a 10-day run-in period to downregulate endogenous FSH, an experimental period of 16 days (Day 11 until Day 26) that included rhFSH dosing on Days 11 and 19, and an end-of-study visit on Day 26.

At screening, gynecological examinations, including breast examination, transvaginal sonography and PAP smear were performed by an experienced gynecologist. Serum FSH levels were determined at screening and at Days 0 and 10. Fourteen ± 3 days into a cycle of OCs (study Day 0), the GnRH agonist (Zoladex^®^ [goserelin acetate implant 3.6 mg, 1-month depot], AstraZeneca GmbH, 22876 Wedel, Germany) was implanted sc to downregulate endogenous FSH [11]. Participants could continue into the treatment period only if downregulation of plasma FSH to <4 IU/L was confirmed 10 days after goserelin administration. Based on previous reports of an initial rise in FSH and luteinizing hormone levels after beginning downregulation [12], participants took their own OCs for another 4–7 days to ensure pregnancy prevention. Participants had to discontinue concomitant prescribed and over-the-counter medications 4 weeks and 2 weeks before XM17 or Gonal-f^®^ administration, respectively, until the final study visit. Grapefruit- and quinine-containing products were not permitted within 72 h before XM17 or Gonal-f^®^ administration to the completion of the end-of-study visit. Participants had to abstain from drinking alcoholic beverages for 72 h both before the screening visit and before Day 0 and from Day 11 to the completion of the end-of-study visit.

All participants fasted for 10 h before rhFSH dosing and until 2 h after dosing. Participants resided in the clinic from the evening before dosing (Days 10 and 18) until the morning of Days 13 and 21, respectively. All other study visits occurred as outpatient visits.

XM17 and Gonal-f^®^ were administered in a two-way crossover design on Days 11 and 19. On the morning of Day 11 (PK period 1), participants received either a single 300-IU sc dose of XM17 or a single 300-IU sc dose of Gonal-f^®^ (according to the randomization scheme), followed by the same sets of participants receiving single doses of Gonal-f^®^ and XM17, respectively, on the morning of Day 19 (PK period 2).

Blood sampling for PK of XM17 and Gonal-f^®^ was performed 10 min predose on Days 11 and 19, then at 0.5, 1, 2, 4, 6, 8, 10, 12, 16, 24, 36, 48, 72, 96, 120, 144 and 168 h postdose. Participants were monitored predose and at regular intervals postdose for ECG and vital signs (pulse rate and BP; temperature was also taken but predose only). Routine laboratory testing was performed on Days 11, 12, 19, 20 and 26. Blood was sampled to monitor for XM17 or Gonal-f^®^ antibodies on Days 11, 19 and 26. A second gynecological exam was performed at the Final Study Visit (Day 26) immediately after which participants were to resume taking OCs. Excluding screening, the study duration was about 27 days per participant.

### Drug product and drug concentration measurements

XM17, produced and tested according to Good Manufacturing Practice and relevant European Pharmacopoeia monographs, was provided in vials of 1.0 mL containing 600 IU XM17 and administered at a dose of 300 IU XM17, which corresponded to an injection volume of 0.5 mL. Commercially available Gonal-f^®^ was provided in pens with integrated vials of 0.5 mL containing 300 IU Gonal-f^®^. During screening and on days 0 and 10, endogenous FSH levels were determined at Medizinisches Versorgungszentrum (MVZ) für Laboratoriumsmedizin und Mikrobiologie GbR (Ulm, Germany) using an electrochemiluminescent immunoassay (ECLIA; Elecsys testkit, Roche Diagnostics, Indianapolis, IN, USA). All PK samples and downregulated endogenous FSH (measured predose on Days 11 and 19) were measured by a validated, double-sandwich, bioanalytical method (MSD^®^ platform) at MDS Pharma Services Switzerland AG. Serum anti-FSH antibodies were determined by ECLIA at MDS Pharma Services Switzerland AG.

## Data analyses

The sample size calculation was based on an assumption of an analysis of variance-coefficient of variation (ANOVA-CV) of 25 % and an expected geometric mean ratio of 1.05 for the PK parameters AUC_0-t_ and C_max_. With a sample size per sequence group of 18 (total sample size of 36), the crossover design had 90 % power to reject both null hypotheses that the test to reference geometric mean ratio was either <0.8 or >1.25 (ie, the test and reference were not equivalent) [7]. PK variables were calculated using noncompartmental methods using WinNonlin^®^ software (Pharsight Version 5.21, Pharsight Corporation, Mountain View, CA, USA). Primary PK parameters were based on actual times and values (ie, not imputed if missing) derived after subtracting predose (baseline) endogenous FSH serum concentrations from values immediately predosing. If subtraction produced a value <0, that value was set to 0. Serum concentrations of XM17 and Gonal-f^®^ below the lower limit of quantification (LLOQ) were labeled as (BLQ) in the serum study drug concentration data listings and set to 0 if recorded predose and to 1/2 LLOQ otherwise. λ_Z_ was calculated by linear regression methods using ≥3 of the last data points above the LLOQ from log-concentration vs time plots. The λ_Z_ (and parameters derived from λ_Z_) were considered sufficiently reliable only if the adjusted r^2^ ≥ 0.85 and were not used otherwise for pharmacometrics. The V_z_/F during the terminal phase was determined as CL/F ÷ λ_Z_.

For the majority of PK parameters (ie, AUCs, C_max_, t_1/2_ and t_max_), descriptive summary statistics were presented by treatment, including n, arithmetic mean, standard deviation (SD), maximum, median, minimum and CV%. All values except t_max_ were corrected for predose FSH concentrations. Statistical differences in PK parameters (except for t_max_) were evaluated using ANOVA mixed-effects procedure on log-transformed values and back-transformed for presentation. Ratios of PK parameters for XM17:Gonal-f^®^ were derived from least squares geometric means (nonparametric Hodges-Lehman evaluation for t_max_). Differences between treatments were considered clinically irrelevant if the 90 % CI was fully contained within the interval between 0.8 and 1.25, the customary range for investigations of bioequivalence [7].

Safety and tolerability data were descriptive (coded using Medical Dictionary for Regulatory Activities [MedDRA] v12.0) but were not evaluated statistically. AEs occurring between Days 0 and 10 were considered TEAEs for goserelin. AEs occurring or worsening after administration of the study drugs on Days 11 or 19 were considered TEAEs for the study drug administered on that respective day. AEs that occurred during PK period 1 (Day 11-18) and that lasted until PK period 2 (Day 19-26), but that did not increase in severity, were counted only for the study drug administered on Day 11. TEAEs occurring on Days 0 through 10 and continuing after administration of study drugs on Days 11 or 19, with increase in severity, were recorded as two events.

## Results

### Participants

**Fig. 1:**
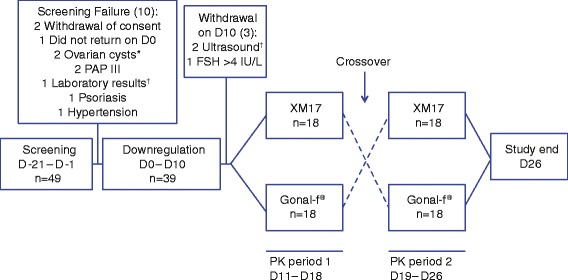
Subject Disposition. ^*^Or clinically relevant findings in transvaginal ultrasound. ^†^Clinically relevant findings

A total of 36 healthy women (mean age 27.1 ± 6.2 years [SD] and BMI 22.7 ± 2.5 kg/m^2^ [SD]) whose endogenous FSH was downregulated to <4 IU/L after a single dose of goserelin were eligible and randomly assigned to treatment (Fig. 1). By the end of the two-way crossover phase of the study (18 participants in each sequence group), all 36 participants had received single doses of XM17 and Gonal-f^®^ and completed the PK and safety evaluations per protocol. Three participants who received only goserelin and did not enter the crossover treatment phase of the study (2 women due to findings on ultrasound examination and 1 woman due to FSH >4.0 IU/L) were included in the safety analysis only. Except for one participant of mixed Caucasian/Hispanic ethnicity, all participants were Caucasian. All were nonsmokers or moderate smokers with negative drug or alcohol screens; none had any clinically significant medical histories or conditions.

### Pharmacokinetics of XM17 compared to Gonal-f^®^

**Fig. 2:**
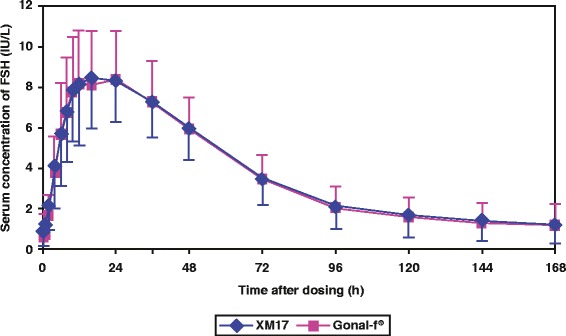
Mean (± SD) serum concentrations of FSH (IU/L) after single sc doses of XM17 or Gonal-f^®^ (corrected for predose FSH levels) in healthy women (linear scale)

Mean (± SD) predose-corrected, serum concentration-time profiles after single 300-IU sc doses of XM17 and Gonal-f^®^ were essentially superimposable (Fig. 2 [linear scale]). Mean PK parameters comparing XM17 and Gonal-f^®^ are listed in Table 1. λ_Z_ was reliably calculable in 15 participants (see Methods); the parameters derived from λ_Z_ could only be calculated in these participants. Ratios of the primary PK parameters (geometric means of XM17:Gonal-f^®^) equaled 1.028 (90 % CI: 0.931, 1.134) for AUC_0-t_ and 1.017 (0.958, 1.080) for C_max_ and fully contained within the prespecified interval (0.8, 1.25) (Table 2). Ratios of the secondary PK parameters, AUC_0-168h_ and AUC_0-∞_, also totaled close to 1 with 90 % CIs again within the prespecified interval. For t_1/2,_ the ratio between the two treatments was 0.939 with a 90 % CI of 0.711 and 1.240. There was almost no difference in t_max_ between XM17 and Gonal-f^®^ (mean t_max_ values 19.78 h and 19.72 h, respectively and median t_max_ 16.0 h for both). No significant sequence or period effects were observed (*P* > 0.1 for all parameters).

**Table 1:** Primary and secondary PK parameters of XM17 and Gonal-f^®^

PK parameter^a^	Statistic	XM17 (*n* = 36)	Gonal-f^®^ (*n* = 36)
AUC_0-t_ [IU∙h/L]	n	36	36
	Mean (SD)	632.0 (167.1)	619.2 (191.1)
	Geom. mean	605.3	589.0
	Median	647.5	598.5
	Min-Max	175–902	261–1131
C_max_ [IU/L]	n	36	36
	Mean (SD)	9.18 (2.42)	9.04 (2.49)
	Geom. mean	8.85	8.70
	Median	9.07	8.81
	Min-Max	3.42–13.99	4.42–14.44
AUC_0-168h_ [IU∙h/L]	n	36	36
	Mean (SD)	632.3 (167.0)	620.3 (189.8)
	Geom. mean	605.7	590.7
	Median	650.5	598.5
	Min-Max	176–902	264–1131
AUC_0-∞_ [IU∙h/L]	N_calc_	15	15
	Mean (SD)	665.6 (179.4)	620.3 (208.6)
	Geom. mean	641.2	583.6
	Median	647.4	622.9
	Min-Max	333–940	268–979
λ_Z_ [1/h]	N_calc_	15	15
	Mean (SD)	0.0258 (0.0164)	0.0239 (0.0131)
	Geom. mean	0.0229	0.0218
	Median	0.0201	0.0190
	Min-Max	0.0134–0.0736	0.0143–0.0663
t_max_ [h]	N_calc_	36	36
	Mean (SD)	19.78 (8.36)	19.72 (7.92)
	Geom. mean	NA^b^	NA^b^
	Median	16.00	16.00
	Min-Max	10.0–48.0	10.0–36.0
t_1/2_ [h]	N_calc_	15	15
	Mean (SD)	32.80 (11.12)	33.73 (10.24)
	Geom. mean	30.32	31.77
	Median	34.43	36.50
	Min-Max	9.41–51.54	10.45–48.38
CL/F [mL/min]	N_calc_	15	15
	Mean (SD)	8.13 (2.58)	9.20 (3.91)
	Geom. mean	7.80	8.57
	Median	7.72	8.03
	Min-Max	5.32–15.02	5.11–18.66
V_z_/F [L]	N_calc_	15	15
	Mean (SD)	21.72 (7.00)	24.38 (6.52)
	Geom. mean	20.47	23.56
	Median	19.68	21.77
	Min-Max	6.7–36.9	13.8–35.7

*AUC*_*0-168h*_ area under the concentration-time curve from time 0–168 h postdose, *AUC*_*0-∞*_ area under the concentration-time curve from time 0 to infinity, *AUC*_*0-t*_ area under the concentration-time curve from time 0 to the time of last observed concentration, *CL/F* apparent clearance, *C*_*max*_ maximum concentration, *λ*_*Z*_ apparent terminal rate constant, *Geom. Mean*, geometric mean, *n* number of participants within group, *NA* not applicable, *N*_*calc*_ number of participants used for calculation, *PK* pharmacokinetic, *SD* standard deviation, *t*_*max*_ time at which C_max_ occurred, *t*_*1/2*_ apparent half-life of terminal elimination, *V*_*z*_ */F* apparent volume of distribution during the terminal phase after extravascular administration

^a^All values are corrected for predose FSH except for t_max_

^b^Geometric mean is not meaningful for t_max._

**Table 2:** Statistical comparison of PK parameters^a^ for XM17 and Gonal-f^®^

Parameter	Ratio of XM17:Gonal-f^®^	90 % CI
AUC_(0-t)_	1.028	(0.931, 1.134)
C_max_	1.017	(0.958, 1.080)
AUC_(0-168h)_	1.025	(0.931, 1.130)
AUC_(0-∞)_	0.997	(0.879, 1.131)
t_1/2_	0.939	(0.711, 1.240)
t_max_, h	0.00 (difference)	(-4.00, 3.00)

*AUC*_*0-168h*_ area under the concentration-time curve from time 0 to 168 h postdose, *AUC*_*0-∞*_ area under the concentration-time curve from time 0 to infinity, *AUC*_*0-t*_ area under the concentration-time curve from time 0 to the time of last observed concentration, *CI* confidence interval, *C*_*max*_ maximum concentration; *PK* pharmacokinetic, *t*_*max*_ time at which C_max_ occurred, *t*_*1/2*_ apparent half-life of terminal elimination

^a^All PK parameters are corrected for predose FSH except for t_max_

### Safety

During the crossover period, 25 (69.4 %) and 20 (55.6 %) participants experienced TEAEs after receiving XM17 and Gonal-f^®^, respectively (Table 3). The TEAEs occurring in all 39 participants after goserelin implantation but before administration of XM17 or Gonal-f^®^ are also included in Table 3 but were not included in the main safety assessment of XM17. Headache was the most common TEAE (29 events in 17 participants overall), followed by hot flushes (10 events in 10 participants overall). In general, TEAEs occurred in similar frequency after both treatments.

**Table 3:** Treatment-emergent adverse events occurring in ≥5 % after goserelin, XM17 or Gonal-f^®^

Event term	Goserelin	XM17	Gonal-f^®^
	(*n* = 39)	(*n* = 36)	(*n* = 36)
	x (y, z %)	x (y, z %)	x (y, z %)
Overall	26 (17, 43.6 %)	43 (25, 69.4 %)	42 (20, 55.6 %)
Headache	11 (10, 25.6 %)	15 (14, 38.9 %)	14 (11, 30.6 %)
Hot flushes	–	5 (5, 13.9 %)	5 (5, 13.9 %)
Abdominal pain, lower	–	1 (1, 2.8 %)	3 (3, 8.3 %)
Nausea	–	2 (2, 5.6 %)	2 (2, 5.6 %)
Dizziness	2 (2, 5.1 %)	2 (2, 5.6 %)	2 (2, 5.6 %)
Metrorrhagia	–	2 (2, 5.6 %)	1 (1, 2.8 %)
Dysgeusia	–	1 (1, 2.8 %)	2 (2, 5.6 %)
Rash	–	1 (1, 2.8 %)	2 (2, 5.6 %)
Injection site pain	–	–	2 (2, 5.6 %)^a^
Abdominal pain	2 (2, 5.1 %)	1 (1, 2.8 %)	1 (1, 2.8 %)
Breast pain	2 (2, 5.1 %)	–	–
Oropharyngeal pain	2 (2, 5.1 %)	1 (1, 2.8 %)	–

^a^These AEs occurred immediately upon injection/drug administration and lasted only one minute each and thus were included in the AE assessment but not the first local tolerability assessment (1 h post-dose). Abbreviation: x (y, z %) = number of events (number of participants, percent by treatment)

Overall, the severity of all TEAEs was mainly mild (39 in 23 [63.9 %] participants) or moderate (45 in 22 [61.1 %] participants). After Gonal-f^®^, one participant experienced dizziness that was considered severe. Moderate TEAEs occurred in a similar frequency during both treatments: 23 events were reported by 15 participants (41.7 %) after XM17 and 22 events were reported by 15 participants (41.7 %) after Gonal-f^®^. There were no serious events and no AEs leading to withdrawal after the start of treatment with either XM17 or Gonal-f^®^. Most TEAEs were judged by the investigator as being “unlikely” or “not related” (53 TEAEs) to XM17 or Gonal-f^®^, while 32 TEAEs were considered possibly related (for XM17, 14 events in 9 [25.0 %] participants; for Gonal-f^®^, 18 events in 9 [25.0 %] participants). No TEAEs were considered to be “probably” related to study drug.

No clinically relevant changes in clinical chemistry, hematology, vital signs or ECG were noted, and no participant tested positive for anti-FSH antibodies. There were no noteworthy physical findings except for one participant with moderate exanthema, which was observed on the abdomen at the end of the study (Gonal-f^®^ had been administered in the second treatment period).

Local tolerability, assessed immediately after XM17 and Gonal-f^®^ dosing and again at 1, 6 and 24 h after dosing, was unremarkable, except for mild hematoma at 24 h after dosing in four participants taking Gonal-f^®^ and mild pain on movement in one participant taking XM17.

## Discussion

The patents on many biologic agents are approaching their expiration dates. However, newer biologics that mimic the innovator product have been slow to develop. The incentive to develop such agents has been hampered by the challenges of manufacturing and extracting from living cells an exact copy of the desired therapeutic protein. The EMA has issued guidance documents regarding the regulatory pathway that manufacturers must follow to gain marketing approval for biosimilars [6, 7]. These guidances address the expectations of the EMA about the documentation required to register a biosimilar. The process demands a rigorous demonstration of comparability, with the dossier expected to include information about the biologic’s mechanism of action, route of administration, formulation, PK, potency, indications for use, data to support safety and efficacy, excipients, manufacturing facility, patent protection and any other aspect of the agent relevant to the safety of the drug class.

Follitropin alfa (Gonal-f^®^) is the reference (innovator) product that has been shown to be safe and effective for the treatment of anovulation, for the stimulation of follicular development in women undergoing assisted reproductive technology procedures, and in those with severe luteinizing hormone and FSH deficiency, as well as for spermatogenesis stimulation in men [13]. It is one of the many complex biologic proteins produced by recombinant technology.

This Phase I study achieved its goal of demonstrating significant bioequivalence of single 300-IU sc doses of XM17 to the reference follitropin alfa (Gonal-f^®^) with respect to the primary PK parameters C_max_ and AUC_0-t_. In addition, all secondary PK parameters were comparable to the reference product. There was no difference in t_max_, and the ratios of C_max_ and AUCs were close to 1 and well contained within the predefined interval of 0.8–1.25, which is the commonly accepted interval required to demonstrate equivalence of two biological products [7]. For the facultative additional parameter t_1/2_, the ratio between the two treatments was 0.939, with a 90 % CI of 0.711 and 1.240. Mean concentration-time profiles after doses of XM17 and Gonal-f^®^ were almost identical.

The systemic and local tolerability of XM17 was also similar to that of approved follitropin alfa [13]. Headache was the most frequently reported AE for both XM17 and Gonal-f^®^. Except for mild pain on movement in one participant, XM17 was well tolerated locally. There were no safety concerns or treatment-related findings from the examination of vital signs, ECG, laboratory or physical examination parameters. No anti-FSH antibodies were detected in this single-dose study, however it should be noted that antidrug antibodies might be more likely to show up with repeated dosing.

## Conclusions

Based on the primary PK parameters measured, single 300-IU sc doses of XM17 (Ovaleap^®^; follitropin alfa, Teva Pharmaceuticals Europe B.V., Utrecht, The Netherlands) and Gonal-f^®^ administered to healthy young women can be considered bioequivalent. The safety and tolerability profile of XM17 was comparable to that of Gonal-f^®^. Ovaleap^®^, an EMA-approved biosimilar to Gonal-f^®^, adds to the choices of infertility products for women undergoing assisted reproductive technology procedures.

## Abbreviations

λ_Z_: apparent terminal rate constant
ANOVA-CV: analysis of variance-coefficient of variation
AUC: area under the serum concentration vs time curve
AUC_0-∞_: area under the serum concentration vs time curve extrapolated to infinity and estimated from predose corrected data
AUC_0-168h_: area under the serum concentration vs time curve from time 0 to 168 h and estimated from predose corrected data
AUC_0-t_: area under the serum concentration vs time curve from time 0 to the last concentration observed above predose levels
BLQ: below the lower limit of quantification
BMI: body mass index
BP: blood pressure
CHO: Chinese hamster ovary
CL/F: apparent clearance
C_max_: maximum predose-corrected serum concentration
ECG: electrocardiogram
ECLIA: electrochemiluminescent immunoassay
EMA: European Medicines Agency
F: fraction absorbed
FSH: follicle-stimulating hormone
GnRH: gonadotropin-releasing hormone
LLOQ: lower limit of quantification
MedDRA: Medical Dictionary for Regulatory Activities
OC: oral contraceptive
PAP smear: Papanicolaou smear
PK: pharmacokinetics
PD: pharmacodynamics
rhFSH: recombinant human follicle-stimulating hormone
t_1/2_: apparent terminal elimination half-life
TEAE: treatment-emergent adverse event
t_max_: time at which C_max_ occurred
V_z_/F: apparent volume of distribution during the terminal phase
